# NeoTCRseek: an integrated platform for high-sensitivity identification of neoantigen-specific TCR clonotypes to track clinical T-cell dynamics

**DOI:** 10.3389/fimmu.2026.1674427

**Published:** 2026-04-22

**Authors:** Bin Song, Geng Liu, Bo Li, Yong Hou, Leo J. Lee

**Affiliations:** 1 College of Life Sciences, University of Chinese Academy of Sciences, Beijing, China; 2 BGI GenoImmune Therapeutics Inc., Wuhan, China; 3 Department of Electrical and Computer Engineering, Donnelly Centre for Cellular and Biomolecular Research, University of Toronto, Toronto, ON, Canada

**Keywords:** neoantigen-specific TCR clonotypes, TCR sequencing, TCR clustering, low-frequency clonotypes, longitudinal T-cell tracking, personalized cancer immunotherapy

## Abstract

**Introduction:**

Identifying neoantigen-specific T-cell receptor (neoTCR) clonotypes is crucial for tracking clinical T-cell dynamics in personalized cancer immunotherapy. Despite advances in experimental and computational approaches for identifying antigen-specific TCR (asTCR) clonotypes, the identification of neoTCR clonotypes remains challenging due to their low frequencies and the limited sensitivity of current methods.

**Methods:**

We introduce NeoTCRseek, an integrated platform that combines extended T-cell culture, deep TCR sequencing, and advanced TCR-clustering tools to enhance neoTCR clonotype identification. NeoTCRseek was developed using a model cytomegalovirus (CMV) antigen and subsequently validated under two neoantigen setups: a single neoantigen and a neoantigen pool representing a multi-antigen context. For each antigen setup, we used three cell sorting-based methods to detect enriched clonotypes and built a validation dataset for asTCR clonotype characterization and NeoTCRseek performance evaluation.

**Results:**

NeoTCR clonotypes exhibited a significantly higher proportion of low-frequency clonotypes (0.01%–0.1%) than CMV-specific ones (70.3% vs. 33.3%). Nonetheless, NeoTCRseek achieved a detection limit of 0.01% and high accuracy in both single- and multi-antigen contexts by integrating expanded clonotype detection with co-clustering-based TCR prediction. Compared with the benchmark, NeoTCRseek improved the mean F1 score across the two neoantigen setups from 0.21 to 0.41.

**Conclusion:**

NeoTCRseek achieves high analytical sensitivity and accuracy in neoTCR clonotype identification and supports multi-antigen analysis, providing an integrated platform for neoTCR clonotype characterization and for tracking clinical T-cell dynamics in personalized cancer immunotherapy.

## Introduction

Identifying neoantigen-specific T-cell receptor (neoTCR) clonotypes is essential for advancing personalized cancer immunotherapy (1–4), as it characterizes anti-tumor T-cell responses and supports therapeutic TCR discovery, T-cell dynamics tracking, and biomarker development (5–10). Neoantigen-specific non-engineered adoptive T-cell therapies, including those using tumor-infiltrating lymphocytes (TILs) or peripheral blood lymphocytes (PBLs), have demonstrated promising potential in refractory solid tumors. Clinical development of these therapies requires highly sensitive neoTCR clonotype detection in T-cell products to enable longitudinal tracking of transferred T-cell dynamics for pharmacokinetic monitoring (11–14). Bulk TCR sequencing (TCR-seq) provides high-throughput profiling, extensive repertoire coverage, and cost-effectiveness in this context (15–17). Numerous TCR-seq-based methods have been developed to identify antigen-specific TCR (asTCR) clonotypes (18, 19). However, identifying neoTCR clonotypes in primary T cells remains challenging because they constitute only a minute fraction of the overall TCR repertoire and each clonotype occurs at extremely low frequency (9, 20). Despite antigen-driven clonal expansion, neoTCR clonotypes may remain infrequent due to their low precursor frequencies and bystander proliferation (18, 21). Nonetheless, their identification remains essential due to their potential for *in vivo* expansion and therapeutic efficacy (21, 22), thereby necessitating highly sensitive detection methods.

Bulk TCR-seq-based methods for asTCR clonotype detection typically combine an antigen-specific T-cell assay and sequencing to detect clonotypes enriched by antigen-specific binding or expanded through antigen-driven proliferation (6, 23). Peptide–MHC (pMHC) multimer sorting directly validates TCR specificity, but is limited by pMHC diversity and low-affinity pMHC–TCR interactions (24, 25). In contrast, cell culture-based methods facilitate signal amplification from low-input cell populations but may introduce expansion bias (26, 27). In parallel, computational approaches for asTCR prediction, including supervised classification and unsupervised clustering, have seen substantial advances in recent years (28, 29). However, key challenges persist, such as the vast diversity of TCR recognition modes, limited training data (especially for supervised methods), and poor generalizability to novel (unseen) epitopes (30–32). Despite these constraints, TCR-clustering tools offer frequency-agnostic insights by exploiting sequence motifs shared among TCRs recognizing the same antigen (33, 34). Therefore, integrating cell culture-based methods with TCR-clustering tools provides a promising strategy for asTCR clonotype identification by leveraging both clonal expansion and sequence similarity (23).

Current methods for neoTCR clonotype identification in clinical settings are constrained by three critical limitations: (1) inadequate sensitivity for detecting clonotypes below 0.1% frequency, since most methods are optimized for viral antigens or high-frequency clonotypes (≥0.1%); (2) unvalidated accuracy owing to the scarcity of well-characterized validation datasets; and (3) limited capacity for simultaneous multi-antigen detection, essential for resolving heterogeneous T-cell responses necessary to combat tumor heterogeneity. Collectively, these challenges underscore the urgent need for approaches tailored to the distinctive characteristics of neoTCR clonotypes.

To address these limitations, we developed NeoTCRseek, an integrated experimental and computational platform designed to enhance the performance of clinical neoTCR clonotype identification. NeoTCRseek incorporates three key innovations: (1) a neoantigen-specific approach enabling detection of clonotypes at frequencies as low as 0.01%; (2) initial validation using datasets derived from three independent validation methods; and (3) evaluation of multi-antigen analysis capabilities using a neoantigen pool. We demonstrate that NeoTCRseek achieves a detection limit of 0.01% and high accuracy in both single- and multi-antigen contexts, thereby establishing an integrated platform for clinical neoTCR clonotype identification in personalized cancer immunotherapy.

## Materials and methods

### Samples and peptides

Peripheral blood mononuclear cells (PBMCs) were obtained from a healthy HLA-A*02:01–positive donor through Shanghai AoNeng Biotechnology Co., Ltd. (Shanghai, China). Three antigen setups were used for peptide-stimulated T-cell culture: (1) a cytomegalovirus (CMV) pp65-derived NV9 antigen (CMV-pp65, NLVPMVATV), (2) a *TWISTNB*-derived neoantigen (Neo-74, KLMGIVYKV), and (3) a neoantigen pool (Neo-Mix) composed of Neo-74, a *PGM5*-derived neoantigen (Neo-2, AVGSYVYSV), and a *ZSWIM8*-derived neoantigen (Neo-19, VTFHIPFEV). All antigenic peptides were synthesized by GenScript (Nanjing, China).

### T-cell culture

Peptide-stimulated T-cell cultures were maintained for at least 25 days, as previously described (21). Briefly, CD8^+^ T cells were isolated from PBMCs using CD8 microbeads (Miltenyi Biotec) and then cryopreserved. The remaining peripheral blood lymphocytes (PBLs) were resuspended in dendritic cell (DC) medium (2 × 10^6^ cells/mL) and incubated for 2 hours for adherence. After removing non-adherent cells, GM-CSF (800 IU/mL) and IL-4 (1,000 IU/mL) were added. Cells were incubated for 2 days. On day 3, CD40L (200 ng/mL) and IFN-γ (400 IU/mL) were added. Antigenic peptides were loaded on day 4, and mature DCs were collected on day 5. Mature DCs and thawed CD8^+^ T cells were co-cultured at a 1:4–1:10 ratio in AIM-V medium with 2% autologous serum and IL-21 (30 ng/mL) for 2–3 days. IL-2 (40–50 IU/mL), IL-7 (5–10 ng/mL), and IL-15 (1–2 ng/mL) were added, and cells were subsequently incubated for 8–10 days. These T cells were re-stimulated and incubated for another 8–10 days.

### Antigen-specific CD8^+^ T-cell enrichment

Three cell sorting strategies were employed to enrich antigen-specific CD8^+^ T cells: peptide-MHC (pMHC) tetramer-based fluorescence-activated cell sorting (FACS), CD137-based FACS, and pMHC tetramer-based magnetic-activated cell sorting (MACS).

#### Tetramer-based FACS

Cells were collected, washed, and resuspended in FACS buffer (PBS supplemented with 2% FBS and 2 mM EDTA). pMHC tetramers were generated via ultraviolet-irradiation-mediated peptide exchange using Flex-T™ HLA-A*02:01 Monomer UVX (BioLegend, catalog no. 280004). For each 1 × 10^6^ cells, staining was performed with APC-Cy™7-conjugated anti-human CD3 (BD Biosciences, catalog no. 557832; 5 μL), Alexa Fluor^®^ 700-conjugated anti-human CD8 (BD Biosciences, catalog no. 557945; 5 μL), and PE-conjugated pMHC tetramer (0.05 μg) for 15 minutes at 4 °C in the dark. After washing twice with FACS buffer, cells were analyzed using a FACSAria II cell sorter (BD Biosciences) with live cell gating based on 4′,6-diamidino-2-phenylindole (DAPI) exclusion, and CD8^+^pMHC-tetramer^+^ T cells were sorted. Flow cytometry data were analyzed using FlowJo software (Tree Star).

#### CD137-based FACS

Cells were stimulated with the relevant antigenic peptide (1 μg/mL) in HIPP-T009 medium (BIOENGINE, FG0103801) and incubated for 20–24 hours at 37 °C in a humidified incubator with 5% CO_2_. After stimulation, cells were stained with APC-conjugated anti-human CD137 (4-1BB) (BD Biosciences, catalog no. 550890; 10 μL/1 × 10^6^ cells), together with the same anti-CD3 and anti-CD8 antibodies described above, for 15 minutes at 4 °C. Following two washes in FACS buffer and DAPI staining, live CD8^+^CD137^+^ T cells were sorted on a FACSAria II sorter. Data were processed using FlowJo.

#### Tetramer-based MACS

Pre-enriched CD8^+^ T cells were centrifuged and resuspended infimmu.2026.1674427MACS buffer (PBS/EDTA containing 2.5% HSA). For each 1 × 10^7^ cells, 20 μL of MACS buffer and 2 μL of PE-conjugated pMHC tetramers were added, mixed gently, and incubated for 30 minutes at 4 °C in the dark. After incubation, cells were washed, centrifuged, and resuspended in MACS buffer. Next, 15 μL of anti-PE UltraPure microbeads (Miltenyi Biotec) and 50 μL of MACS buffer were added per 1 × 10^7^ cells. The suspension was gently mixed and incubated for 30 minutes at 4 °C in the dark. After a final wash, magnetic separation was performed according to the manufacturer’s instructions, and PE-positive cells were collected for downstream analyses.

### TCR sequencing

The rearranged TCRβ CDR3 regions were amplified and sequenced by BGI-Wuhan (Wuhan, China) using a previously reported genomic DNA (gDNA)-based multiplex PCR method (35). Briefly, gDNA was extracted using the QIAamp DNA Mini Kit (QIAGEN) following the manufacturer’s instructions. A standard input of 500 ng gDNA per sample was amplified using the QIAGEN Multiplex PCR Kit with 30 forward V primers and 13 reverse J primers to target the TCRβ CDR3 regions. The amplified products (100–200 bp) were purified by 2% agarose gel electrophoresis. The purified products were denatured and circularized into single-stranded circular DNA molecules, which were then amplified via rolling circle amplification to generate DNA nanoballs (DNBs). High-quality DNBs were then loaded into patterned nanoarrays and sequenced on the MGISEQ-2000 platform (paired-end, 2 × 100 bp), yielding a default 1 Gbp (gigabase pairs) of data per sample (~10 million reads).

### TCR-seq data processing

The sequencing data were processed using SOAPnuke (v2.1.8), followed by MiXCR (v4.3.2). First, low-quality and adapter-containing reads were filtered from the raw FASTQ files using SOAPnuke with the following parameters: -l 20 -q 0.1 -n 0.01 -f AAGTCGGAGGCCAAGCGGTCTTAGGAAGACAA -r AAGTCGGATCGTAGCCATGTCGTTCTGTGAGCCAAGGAGTTG. The resulting clean reads were processed by MiXCR to align to the reference germline V, D, J, and C gene sequences with the “generic-tcr-amplicon” preset and to assemble TCRβ clonotypes with the “-OaddReadsCountOnClustering = true” parameter enabled. After removing non-functional TCR rearrangements (out-of-frame or containing stop codons) with parameters “--filter-out-of-frames --filter-stops,” final clonotypes were exported and converted to VDJtools format. Clonotypes were ranked by read count, and a summary table was generated listing each clonotype’s read count, frequency, nucleotide and amino acid CDR3 sequences, and assigned V, D, and J genes. Clonotypes with fewer than 10 supporting reads at the nucleotide level were excluded. Shannon index was calculated using VDJtools (v1.2.1) with default settings.

### Jurkat spike-in experiment

The Jurkat E6–1 cell line (ATCC) expresses a monoclonal TCR β-chain with the CDR3 sequence TRBV12-3–CASSFSTCSANYGYTF–TRBJ1-2. Genomic DNA from Jurkat cells was spiked into background DNA from a T-cell-rich, high-diversity (Shannon index = 9.35) T-cell product at absolute clone frequencies of 1%, 0.1%, and 0.01%, creating a dilution series. To verify the expected clonotype frequencies, the spike-in samples were analyzed using two duplex droplet digital PCR (ddPCR) assays (36). Briefly, the T-cell fraction was determined using the unrearranged TCR assay, while the Jurkat cell fraction was measured using a Jurkat-specific assay. The proportion of Jurkat cells within the T-cell population was calculated as the ratio of the Jurkat cell fraction to the T-cell fraction. ddPCR assays were performed by Forevergen (Guangzhou, China). Each dilution was tested in three technical replicates.

### Enriched clonotype detection

Enriched clonotypes were defined by a higher frequency in the antigen-specific population relative to both non-specific and unsorted populations (24, 25). Fold change (FC) is calculated as the ratio of a clonotype’s frequency in the antigen-specific population to its frequency in the non-specific and unsorted populations, respectively. Clonotypes detected in the antigen-specific population but absent in the non-specific population were assigned a pseudo-frequency of 0.0001% in the non-specific population. For CMV-pp65, the FC threshold is set to 1. For neoantigen setups, the threshold is 5 for both pMHC tetramer-based FACS and CD137-based FACS methods, and 1 for pMHC tetramer-based MACS methods. The FC threshold was determined based on the overall enrichment fold from flow cytometry data and the frequency changes of known asTCR clonotypes in TCR sequencing data.

### Expanded clonotype detection

The NeoTCRseek pipeline was implemented using a custom Python script. By default, only clonotypes with a frequency ≥0.01% in the stimulated culture were included in the analyses. FC for each clonotype was calculated as the ratio of its frequency in the peptide-stimulated culture to that in the peptide-free culture (negative control, NC). For clonotypes detected in the stimulated culture but absent from the control culture, a pseudo-frequency of 0.0001% was assigned to the control culture. Fisher’s exact test was performed for each clonotype to compare read counts between the peptide-stimulated culture and the control culture, yielding an odds ratio and p-value. To prevent zero cell counts from producing infinite odds ratio estimates, a pseudo-count of 1 was added to each cell of the 2×2 contingency table prior to testing. False discovery rate (FDR) was calculated using the Benjamini-Hochberg procedure to correct for multiple comparisons based on the p-values obtained from Fisher’s exact test. For benchmarking, the “Fold change only” strategy (FC ≥ 100) was used to define expanded clonotypes. For NeoTCRseek, the “Fold change + NC filter” strategy was applied, combining FC ≥ 300 with an NC-based filter that excluded clonotypes not detected in the NC sample.

### TCR clustering

Three state-of-the-art TCR-clustering tools (GIANA, GLIPH2, and TCR-VALID) were evaluated in this study. Because TCR-VALID lacks a built-in clustering module, DBSCAN (Density-Based Spatial Clustering of Applications with Noise) was applied to perform clustering on its output representations. GIANA (-t 9 -S 1), GLIPH2 (local_min_pvalue = 0.01, kmer_min_depth = 2, local_min_OVE = 8), and TCR-VALID with DBSCAN (eps = 3, min_samples = 2, metric = “manhattan”) were configured with optimized clustering radii and related parameters to maximize clustering performance.

### Performance metrics

Overlap coefficient (OC) measures the overlap between two sets of elements (e.g., clonotype lists) by dividing the size of their intersection by the size of the smaller set (OC(A, B) = |A ∩ B|/min(|A|, |B|)); here, it quantified the fraction of shared clonotypes in the frequency-filtered replicate to evaluate repeatability in TCR-seq clonotype identification.

Cumulative mean coefficient of variation (CMCV, %) computes the average of the coefficient of variation (CV) for a given clonotype and all clonotypes with higher frequency, serving as a metric for repeatability in TCR-seq clonotype quantification. Here, a clonotype’s CV refers to the variation in its frequency between replicate samples.

Cohen’s kappa coefficient (κ) quantifies pairwise agreement beyond chance for categorical items (e.g., “detected” vs. “undetected”); here, it was used to assess concordance among the detection results from different validation methods.

The area under the precision-recall curve (AUPR) measures the trade-off between precision (TP/(TP+FP)) and recall (TP/(TP+FN)) across thresholds, making it especially informative for imbalanced datasets; here, it guided the selection of the optimal filtering metric in the cell culture-based method.

The F1 score (the harmonic mean of precision and recall) was calculated with Scikit-Learn’s built-in ‘f1_score’ function and employed to determine the optimal threshold for the FC-based approach. Additionally, a frequency-weighted F1 score, incorporating clonotype frequencies via the function’s ‘sample_weight’ parameter, was computed to emphasize high-frequency clonotypes.

## Results

### Overview of NeoTCRseek development

**Figure 1:**
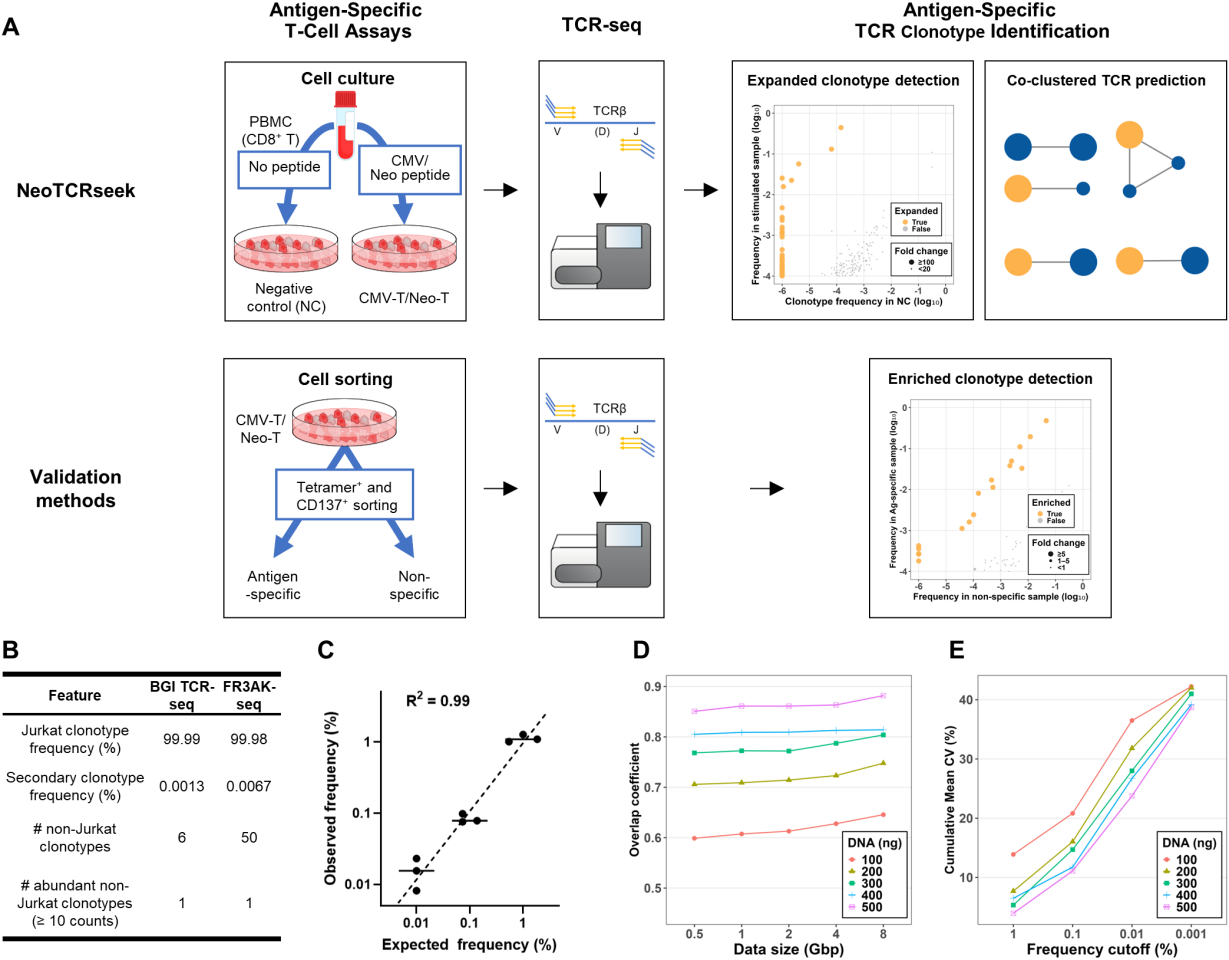
Overview of the NeoTCRseek methodology and evaluation of bulk TCR sequencing performance. **(A)** Schematic illustration of the NeoTCRseek workflow and validation methods. T-cell products stimulated with CMV-pp65 or neoantigen peptides are referred to as CMV-T and Neo-T, respectively. T cells cultured without peptide stimulation served as the negative control (NC). **(B)** Clonotype identification accuracy of DNA-based TCR-seq (BGI TCR-seq), evaluated in a monoclonal Jurkat cell line and benchmarked against an RNA-based TCR-seq method (FR3AK-seq) using triplicate measurements. **(C)** Clonotype quantification accuracy of the DNA-based TCR-seq, evaluated using spike-in standards. R^2^ denotes the square of the Pearson correlation coefficient between observed and expected clonotype frequencies. **(D)** Clonotype identification repeatability of the DNA-based TCR-seq for clonotypes with a frequency ≥0.01%. Overlap coefficients of clonotypes from replicate samples were computed across varying input DNA amounts (ng) and sequencing data sizes (Gbp). **(E)** Clonotype quantification repeatability of the DNA-based TCR-seq. The cumulative mean coefficients of variation (CMCVs, %) of clonotype frequencies in replicate samples were computed across varying input DNA amounts (ng) and clonotype frequency cutoffs.

NeoTCRseek was developed by integrating long-term peptide-stimulated T-cell culture, deep TCR-seq, and advanced TCR-clustering tools, and its performance was validated using three independent methods (**Figure 1A**). To develop and validate this method, we investigated three antigen setups: (1) a cytomegalovirus (CMV) pp65-derived antigen (CMV-pp65), (2) a *TWISTNB*-derived neoantigen (Neo-74), and (3) a neoantigen pool (Neo-Mix) comprising Neo-74 and two additional neoantigens. NeoTCRseek was initially developed using CMV-pp65 and subsequently validated for neoTCR clonotype identification in both single- and multi-antigen contexts.

For each antigen setup, peptide-stimulated T-cell culture was maintained for at least 25 days to drive the exponential expansion of asTCR clonotypes and to generate a T-cell product (21, 27). In parallel, an unstimulated (peptide-free) culture was included as a negative control (NC). Following cell culture, genomic DNA was extracted from both stimulated and control cultures and subjected to deep TCR-seq. Due to the inherent scarcity of experimental replicates in clinical settings, a common approach to identify expanded clonotypes is to assess the significance of clonal expansion in the stimulated culture compared with the NC. This typically involves filtering by clonotype frequency along with either fold change (FC) or odds ratio, followed by Fisher’s exact test (37, 38). For long-term T-cell cultures, previous research recommends a high-stringency threshold of FC ≥ 100 (27). Using this criterion (FC ≥ 100) as a benchmark, we developed NeoTCRseek to accurately detect expanded clonotypes. To leverage TCR sequence information and address the limited accuracy of existing TCR-clustering tools, we introduced a co-clustering approach to predict asTCRs by clustering all TCRs based on sequence similarity and identifying clusters enriched for expanded clonotypes.

To evaluate NeoTCRseek’s accuracy, we built a validation dataset by detecting asTCR clonotypes in the T-cell product for each antigen setup using three cell sorting-based methods: pMHC tetramer-based fluorescence-activated cell sorting (FACS), pMHC tetramer-based magnetic-activated cell sorting (MACS), and activation-induced marker (CD137)-based FACS. Each method uses distinct labeling and isolation strategies to ensure robust benchmarking (39). Previously generated T-cell products were sorted into antigen-specific and non-specific populations, and TCR-seq was performed for each. Enriched clonotypes were defined as those whose frequencies in the antigen-specific population exceeded those in both the non-specific and unsorted populations. Clonotypes enriched with high confidence by the three validation methods were designated “validated asTCR clonotypes” (positives), while all remaining clonotypes were considered non-specific (negatives).

### Optimization of DNA-based deep TCR sequencing for low-frequency clonotype detection

To establish the cell culture-based component of NeoTCRseek, we utilized a DNA-based deep TCR-seq approach for sensitive and accurate clonotype profiling (15, 40). Although this method has been benchmarked before (41), its performance in detecting low-frequency clonotypes (0.01%–0.1%) in highly diverse repertoires remains poorly characterized. In particular, while substantial input material and deep sequencing are required to achieve adequate coverage (15), the exact conditions necessary for reliable detection remain undefined (42). To address these gaps, we systematically evaluated the accuracy and repeatability of our approach for detecting low-frequency clonotypes, including evaluations using a high-diversity sample (Shannon index = 9.35).

Accuracy was first evaluated using defined reference samples. In the monoclonal Jurkat cell line, any detected non-Jurkat clonotype was classified as a technical artifact attributable to PCR or sequencing error. We used this sample to evaluate clonotype identification accuracy and found that our approach detected the Jurkat clonotype at 99.99% frequency, outperforming the performance claimed by FR3AK-seq (**Figure 1B**), a high-performance RNA-based TCR-seq method (43). Furthermore, using spike-in standards with predefined Jurkat clonotype frequencies (down to 0.01%), we demonstrated that our approach achieved near-perfect linearity between observed and expected frequencies (R^2^ = 0.99; **Figure 1C**).

Repeatability was then evaluated by varying input DNA amounts (100–500 ng) and sequencing data sizes (0.5–8 Gbp) during TCR-seq to identify optimal conditions. For clonotypes with a frequency of at least 0.01%, increasing data size modestly improved identification repeatability, while increasing input amounts substantially improved the repeatability in both clonotype identification and quantification (**Figures 1D, E**). Using 500 ng of input DNA and 1 Gbp of sequencing data, this approach achieved high repeatability in both clonotype identification (overlap coefficient = 0.86) and quantification (cumulative mean coefficient of variation = 23.77%). Our results demonstrate that this approach can reliably detect low-frequency clonotypes in highly diverse repertoires under defined experimental conditions. This sensitive TCR-seq approach provided a robust foundation for subsequent asTCR clonotype identification.

### Development of the cell culture-based component of NeoTCRseek using CMV-pp65

**Figure 2:**
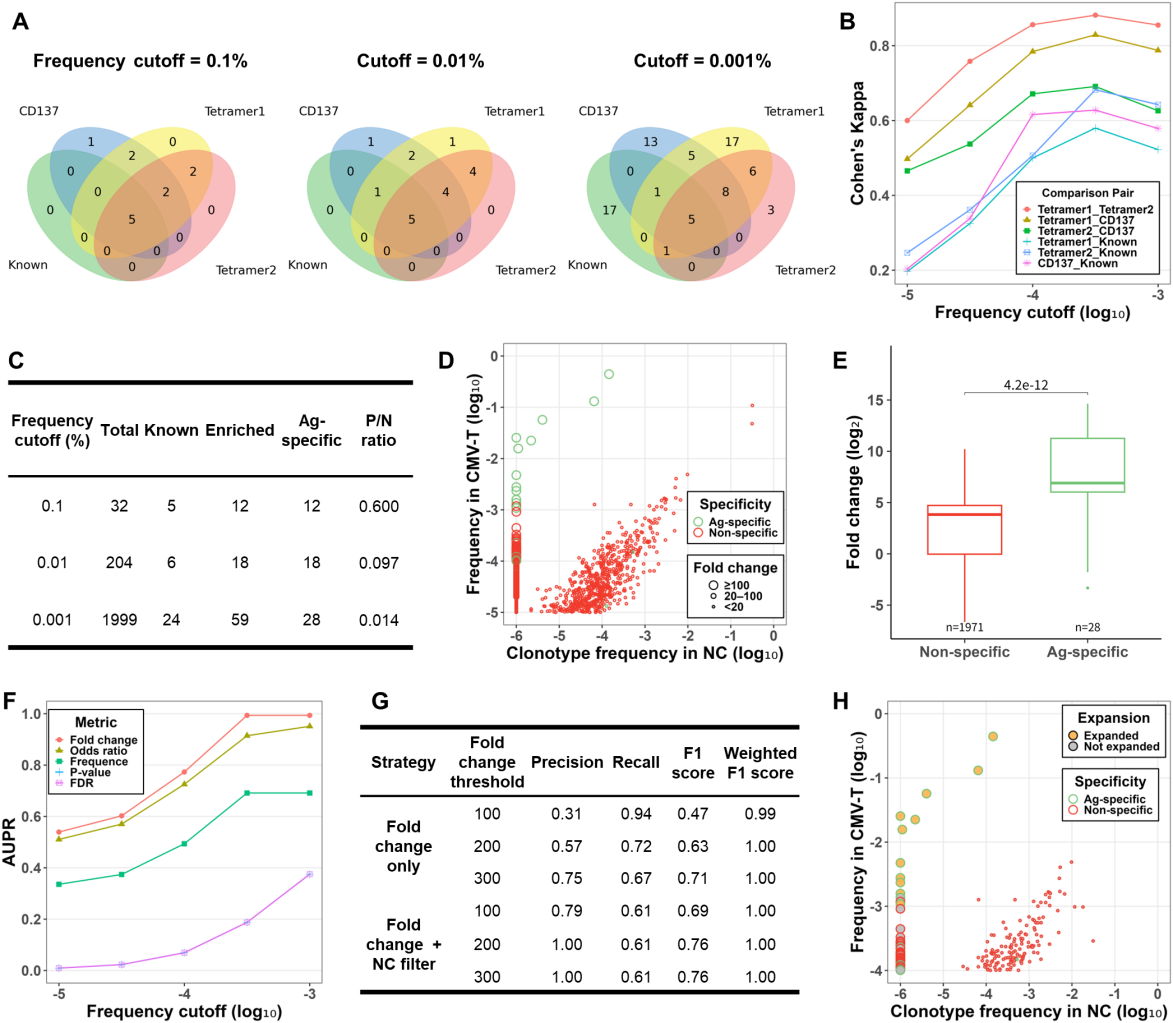
Detection of CMV-specific expanded clonotypes: validation dataset construction, filtering metrics, and method comparison. **(A)** Venn diagrams showing the overlap among four TCR datasets at three clonotype frequency cutoffs: 0.1% (left), 0.01% (middle), and 0.001% (right). The datasets comprise three sets of enriched clonotypes detected by distinct cell sorting-based methods (Tetramer1: pMHC tetramer-based FACS, Tetramer2: pMHC tetramer-based MACS, and CD137: CD137-based FACS), along with one dataset derived from public databases (Known). **(B)** Pairwise agreement among the four datasets at different frequency cutoffs, evaluated using Cohen’s kappa coefficient. **(C)** Clonotype counts for total, known, enriched, and antigen-specific (validated) clonotypes, along with positive-to-negative (P/N) ratios at the three frequency cutoffs. **(D)** Clonotype frequencies in the CMV-T sample and the negative control (NC) sample, with validated clonotypes outlined in green. The size of the points represents the fold change (FC). Only clonotypes with ≥0.001% frequency in CMV-T are shown. **(E)** Comparison of FC between non-specific and antigen-specific clonotypes. P-values were determined by Wilcoxon rank-sum tests. **(F)** Performance of five metrics in detecting antigen-specific TCR (asTCR) clonotypes at different frequency cutoffs, evaluated using the area under the precision-recall curve (AUPR). **(G)** Accuracy of asTCR clonotype detection under different FC thresholds for clonotypes with ≥0.01% frequency. Precision, recall, F1 score, and weighted F1 score were evaluated for two strategies: “Fold change only” and “Fold change + NC filter” (FC combined with NC–based frequency filter). Weighted F1 scores were calculated using clonotype frequencies as sample weights. **(H)** Clonotype frequencies in the CMV-T sample and the NC sample, with detected expanded clonotypes shown as yellow dots and validated clonotypes outlined in green. Only clonotypes with ≥0.01% frequency in CMV-T are shown.

We used CMV-pp65 as a model antigen to develop the cell culture-based component of NeoTCRseek. To build a validation dataset, concordances of enriched clonotypes detected by the three validation methods at different frequency cutoffs were qualitatively visualized using Venn diagrams and quantitatively assessed using Cohen’s kappa coefficients (**Figures 2A, B**). Publicly available CMV-specific TCRs (44–46) were included as known asTCR clonotypes for benchmarking. The results indicated that: (1) at higher frequency cutoffs (0.1% and 0.01%), agreements among the results from the methods were substantial or almost perfect (Cohen’s κ > 0.6), with all six known clonotypes and 12 clonotypes of unknown specificity detected; (2) at the 0.001% cutoff, agreements declined to the moderate range. Among the 18 known clonotypes within the ultra-low-frequency range (0.001%–0.01%), 94.4% (17/18) were undetected by any validation methods. Such consistent detection (or non-detection) across three validation methods provides preliminary evidence for (or against) the antigen specificity of a given TCR clonotype. To further assess the antigen specificity of the enriched clonotypes of unknown specificity and the undetected known clonotypes, we examined their clonal expansion using FC. Notably, 91.7% (11/12) of the enriched clonotypes of unknown specificity exhibited substantial expansion (FC ≥ 100), providing strong evidence that they represent novel asTCR clonotypes. In contrast, 88.2% (15/17) of the undetected known clonotypes had an FC below 20 and were classified as non-specific. These results collectively show that the validation methods accurately detect asTCR clonotypes present at frequencies of 0.01% or higher.

We therefore defined “validated asTCR clonotypes” as those detected at ≥0.01% frequency by any validation method, or at 0.001%–0.01% frequency by at least two methods. Thus, a validation dataset was constructed, comprising clonotypes with a frequency of at least 0.001% (**Figure 2C**). The validation dataset was stratified into multiple subsets based on frequency cutoffs to evaluate the sensitivity of the detection method. Progressively lowering the frequency cutoff reduced the subsets’ positive-to-negative (P/N) ratio (the ratio of antigen-specific clonotypes to non-specific clonotypes) from 0.6 to 0.014, highlighting the increased challenge of detecting asTCR clonotypes at ultra-low frequencies. As expected, the majority (17/18) of antigen-specific clonotypes with a frequency ≥0.01% showed substantial expansion in the stimulated culture (**Figure 2D**). Compared to non-specific clonotypes, antigen-specific ones exhibited significantly higher levels of expansion (*p* = 4.2 × 10^-12^; **Figure 2E**).

Next, we applied five widely used filtering metrics—FC, odds ratio, frequency, Fisher’s exact test p-value, and false discovery rate (FDR)—to subsets of the validation dataset for asTCR clonotype detection and evaluated their performance using the area under the precision–recall curve (AUPR). The FC-based approach outperformed the others across all frequency cutoffs, achieving an AUPR of 0.77 for clonotypes with a frequency ≥0.01% (**Figure 2F**). Due to the large read depth, statistical tests may yield significant p-values even for clonotypes with minimal expansion, thereby reducing precision and lowering the AUPR. Consequently, FC was selected as the primary filtering metric for subsequent method development, without incorporating p-values or FDR-adjusted significance criteria. However, the performance of the FC-based approach declined as the frequency cutoff decreased, consistent with the other metrics. Due to poor performance in identifying asTCR clonotypes at ultra-low frequencies, subsequent analyses were restricted to those with a frequency of at least 0.01%. At this cutoff, the P/N ratio within the subset was 0.097.

We first employed an “FC-only” strategy to detect asTCR clonotypes and found that increasing the FC threshold from 100 to 300 raised the F1 score from 0.47 to 0.71 (**Figure 2G**). To account for variations in clonotype frequency and the greater significance of high-frequency clonotypes, frequencies were incorporated as sample weights in the F1 score calculation (see Methods). The resulting frequency-weighted F1 score approached 1.00, indicating highly accurate detection of high-frequency asTCR clonotypes. Next, we employed an “FC + NC filter” strategy, combining FC with an NC-based filter to exclude clonotypes absent from the NC (frequency = 0). The results showed that the “FC + NC filter” strategy with an FC threshold of 300 yielded the highest F1 score (0.76). We classified any clonotype detected by this method as an “expanded clonotype.” Using this method, 11 expanded clonotypes were detected and all validated as true positives from a total of 204 clonotypes, with the lowest frequency being 0.1% (**Figure 2H**).

### Validation of the cell culture-based component for neoTCR clonotype detection

**Figure 3:**
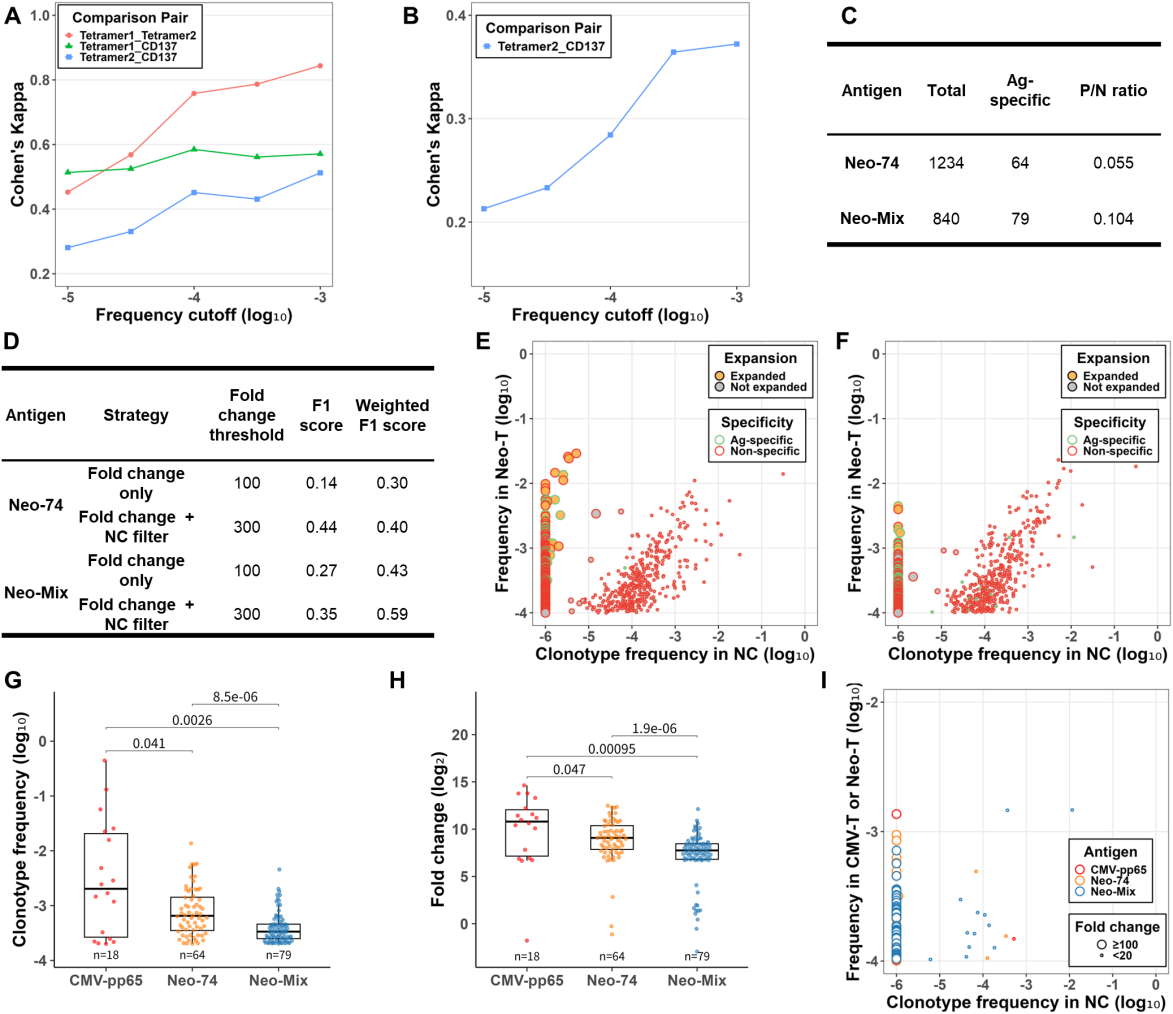
Detection of expanded clonotypes targeting Neo-74 and Neo-Mix: validation dataset construction, method comparison, and performance analysis. **(A, B)** Pairwise agreement (Cohen’s kappa coefficient) among enriched clonotypes targeting **(A)** Neo-74 and **(B)** Neo-Mix across various frequency cutoffs (0.001% to 0.1%). Enriched clonotypes were detected by up to three cell sorting-based methods: Tetramer1 (pMHC tetramer-based FACS), Tetramer2 (pMHC tetramer-based MACS), and CD137 (CD137-based FACS). Note that the pMHC tetramer-based FACS assay (Tetramer1) for Neo-Mix failed due to insufficient CD8^+^ pMHC-tetramer^+^ cells. **(C)** Total clonotype counts, antigen-specific (validated) clonotype counts, and positive-to-negative (P/N) ratios for Neo-74 and Neo-Mix. **(D)** Evaluation of neoTCR clonotype detection accuracy for Neo-74 and Neo-Mix at a ≥0.01% frequency threshold, using F1 and frequency-weighted F1 scores. Performance comparison between the “Fold change only” strategy (FC threshold = 100) and the “Fold change + NC filter” strategy (FC threshold = 300). **(E, F)** Clonotype frequencies in the Neo-T sample and the NC sample for **(E)** Neo-74 and **(F)** Neo-Mix, with expanded clonotypes marked as yellow dots and validated clonotypes outlined in green. Only clonotypes with ≥0.01% frequency in Neo-T are shown. **(G, H)** Distribution of **(G)** Clonotype frequency and **(H)** FC for validated clonotypes across three antigen setups: CMV-pp65 (red, n=18), Neo-74 (orange, n=64), and Neo-Mix (blue, n=79). P-values were determined by Wilcoxon rank-sum tests. **(I)** Frequencies of false-negative clonotypes in the CMV-T or Neo-T sample and the NC sample across the three antigen setups: CMV-pp65 (red), Neo-74 (orange), and Neo-Mix (blue).

Based on the cell culture-based component developed using CMV-pp65, we evaluated its applicability to neoTCR clonotype detection by building validation datasets composed of clonotypes with a frequency of at least 0.01% and evaluating the performance of the final FC-based approach. To build validation datasets, we first assessed the concordance among enriched clonotypes detected by the three validation methods across two neoantigen setups (Neo-74 and Neo-Mix). For clonotypes with a frequency of at least 0.01%, those detected by the two tetramer-based methods showed substantial agreement, whereas their agreements with the CD137-based method were only fair to moderate (**Figures 3A, B**). To ensure consistency with CMV-pp65, we defined validated asTCR clonotypes as those detected by any validation method and built corresponding validation datasets (**Figure 3C**). The P/N ratios for the datasets of Neo-74 and Neo-Mix were 0.055 and 0.104, respectively.

Next, we evaluated the performance of the final FC-based approach for neoTCR clonotype detection (**Figure 3D**). For Neo-74, the final FC-based approach achieved an F1 score of 0.44, significantly outperforming the benchmark of 0.14. Among 1,234 clonotypes, 92 were detected as expanded, including 34 validated as true positives; notably, the lowest detectable frequency among these clonotypes was 0.03% (**Figure 3E**). For Neo-Mix, the final FC-based approach achieved an F1 score of 0.35, exceeding the benchmark of 0.27. Among 840 clonotypes, 30 were detected as expanded, with 19 validated as true positives, with the lowest frequency being 0.03% (**Figure 3F**).

Our method outperformed the benchmark. However, despite using the same approach, neoTCR clonotype detection was less accurate than asTCR clonotype detection for the CMV-pp65 antigen. The P/N ratio in the Neo-Mix dataset was slightly higher than that of CMV-pp65, indicating that it was not the main factor. However, the overall frequency of asTCR clonotypes was significantly lower in neoantigen datasets than in the CMV-pp65 dataset (*p* < 0.05, Wilcoxon rank-sum test) (**Figure 3G**). Specifically, the proportion of low-frequency clonotypes among asTCR clonotypes was significantly higher in the Neo-74 (70.3%) and Neo-Mix (89.9%) datasets than in the CMV-pp65 dataset (33.3%). Beyond clonotype frequency, the overall fold-change magnitude of asTCR clonotypes was significantly lower in the neoantigen datasets than in the CMV-pp65 dataset (**Figure 3H**), further complicating the distinction between true expanded clonotypes and background noise. While the FC threshold for neoantigen datasets could be adjusted, such optimization would yield more robust insights if validated with additional independent datasets.

These findings suggest that improving the identification strategy is essential for enhancing performance. One potential approach to reduce false positives is to exclude clonotypes that also expand in response to non-target antigens. For Neo-74, only one false-positive asTCR clonotype exhibited substantial expansion in response to CMV-pp65 (FC = 194), suggesting that cross-reactivity accounted for a minority of false-positive events in this setting. To better characterize false negatives, we examined their expansion profiles and found that most exhibited substantial expansion but did not reach the 300-fold threshold used in the initial screening. Specifically, across the three antigen setups, 85.7% (6/7), 90.0% (27/30), and 80.0% (48/60) of the false-negative asTCR clonotypes fell within this intermediate expansion range (**Figure 3I**). These findings suggest that the stringent threshold, while maximizing the F1 score, may have reduced sensitivity for moderately expanded yet asTCR clonotypes, motivating the development of a targeted rescue strategy.

### A co-clustering approach for predicting neoTCRs

**Figure 4:**
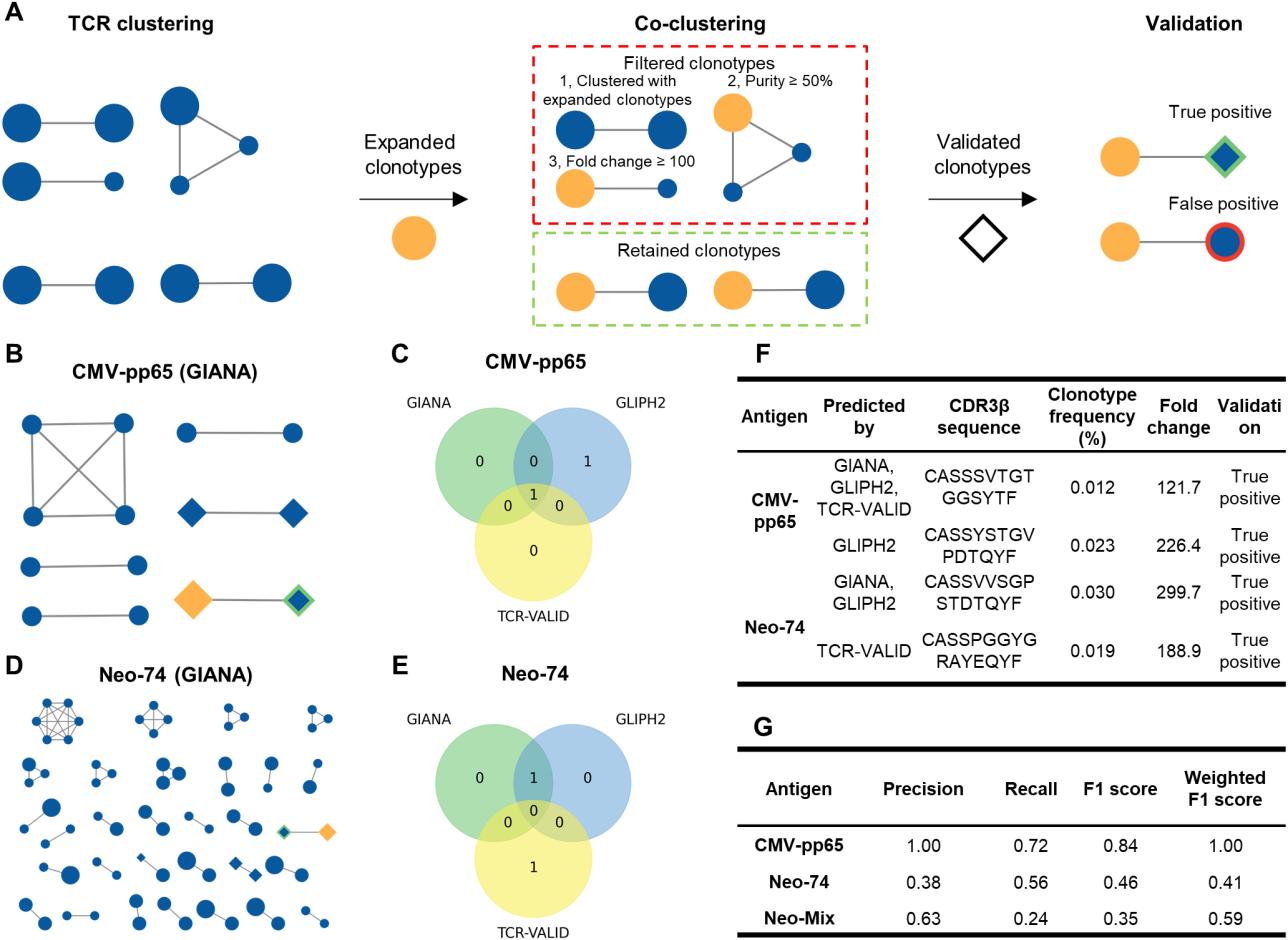
Prediction and validation of co-clustered TCRs. **(A)** Schematic overview of the workflow used for predicting and validating co-clustered TCRs. **(B)** Co-clustered TCRs predicted by GIANA for CMV-pp65. **(C)** Overlap of predicted co-clustered TCRs across GIANA, GLIPH2, and TCR-VALID tools for CMV-pp65. **(D)** Co-clustered TCRs predicted by GIANA for Neo-74. **(E)** Overlap of predicted co-clustered TCRs across GIANA, GLIPH2, and TCR-VALID tools for Neo-74. **(F)** Predicted co-clustered TCRs for CMV-pp65 and Neo-74, including prediction tools, CDR3β sequences, clonotype frequency (%), fold change, and validation status. **(G)** Performance evaluation of asTCR clonotype identification using NeoTCRseek for CMV-pp65, Neo-74, and Neo-Mix, quantified by precision, recall, F1 score, and frequency-weighted F1 score.

To enhance neoTCR clonotype identification, we developed a co-clustering approach to predict asTCRs based on the detected expanded clonotypes (**Figure 4A**). This approach involves two steps: (1) clustering TCRs of all clonotypes and (2) retaining clusters in which at least 50% of clonotypes are expanded, followed by selecting clonotypes exhibiting ≥100-fold expansion within these clusters. Predicted asTCRs overlapping with experimentally validated clonotypes were considered true positives for evaluation.

To test feasibility, we applied the widely used TCR-clustering tool GIANA (31, 47), which identified one validated clonotype for CMV-pp65 (**Figure 4B**). We then evaluated two additional TCR-clustering tools (GLIPH2 (48) and TCR-VALID (49)) to examine whether the recovery of validated clonotypes was reproducible across tools. GLIPH2 and TCR-VALID identified the same validated clonotype as GIANA, while GLIPH2 additionally recovered one more validated clonotype (**Figure 4C**). Although the total number of recovered clonotypes was limited, the partial concordance across tools supports the feasibility of this approach. TCRs identified by this approach were therefore defined as co-clustered TCRs.

We subsequently applied this approach to the Neo-74 dataset. GIANA identified one validated clonotype (**Figure 4D**), GLIPH2 recovered the same clonotype, and TCR-VALID identified an additional validated clonotype (**Figure 4E**). Despite differences in encoding strategies among the three tools, their partially overlapping predictions and consistent recovery of validated clonotypes across antigen datasets provide preliminary support for the utility of the co-clustering approach. Notably, all recovered clonotypes were low-frequency (minimum 0.012%; **Figure 4F**), demonstrating the high sensitivity of the co-clustering approach in identifying asTCR clonotypes.

Based on findings from both clonal expansion and co-clustering analyses, we developed a unified platform, NeoTCRseek, incorporating two key enhancements: (1) long-term T-cell culture combined with DNA-based deep TCR-seq for sensitive detection and (2) integration of expanded clonotype detection with co-clustering-based TCR prediction to further improve analytical sensitivity and accuracy. NeoTCRseek achieved a detection limit of 0.01%, with F1 scores of 0.84 (CMV-pp65), 0.46 (Neo-74), and 0.35 (Neo-Mix). Compared with the benchmark, it improved the mean F1 score of neoTCR clonotype identification from 0.21 to 0.41 (**Figures 3D**, **4G**).

## Discussion

Comparative analyses of our *in vitro* T-cell culture datasets revealed two key clonotypic features: (1) neoantigen-specific TCR repertoires were less oligoclonal than the CMV-specific repertoire; (2) neoTCR clonotypes were predominantly low-frequency. Since the T cells originated from a healthy donor, these features likely reflect recruitment of naïve CD8^+^ T cells in response to neoantigen stimulation, whereas CMV-pp65 exposure drives memory CD8^+^ T-cell expansion (27). Given the low frequency of neoantigen-specific T cells in PBLs and TILs, and the broad TCR diversity resulting from tolerogenic priming (20, 26, 50), T-cell products from cancer patients may retain similarly diverse neoantigen-specific TCR repertoires enriched for low-frequency clonotypes (51, 52). This complicates the reliable identification of neoTCR clonotypes in clinical settings.

Based on these observations, we developed NeoTCRseek, an integrated platform that combines clonotype frequency with TCR sequence information to identify neoTCR clonotypes. We developed an FC-based method to detect expanded clonotypes and validated its performance, demonstrating superior accuracy compared to the benchmark. We also demonstrated that a co-clustering approach could recover low-frequency clonotypes with substantial expansion. In proof-of-concept evaluations, NeoTCRseek demonstrated high sensitivity and improved accuracy in detecting neoTCR clonotypes across both single- and multi-antigen contexts.

Despite its potential, NeoTCRseek has a few limitations. First, its experimental framework presents inherent challenges: (1) bulk TCR-seq lacks information on chain pairing, so the detected asTCR clonotypes represent putative antigen specificity inferred from statistical association, rather than confirmed specificity (18, 53); (2) cell culture protocols require further optimization to reduce bystander proliferation (5, 54); and (3) the impact of experimental variability could be mitigated by incorporating biological or technical replicates and robust statistical methods (55, 56). Second, its generalizability is limited by fixed parameter thresholds and requires validation across broader datasets. Third, the validation presumes that clonotypic enrichment or expansion unequivocally signifies neoTCR specificity; however, this assumption is undermined by the disconnect between TCR binding avidity and downstream T-cell functionality, and is further confounded by intrinsic T-cell functional heterogeneity (57). Consistent with prior work (55), we observed large discrepancies among validation methods, particularly in low-frequency clonotypes. These findings underscore the limitations of approaches that rely on a single T-cell characteristic and highlight the advantages of integrated, multi-parameter approaches. However, scaling such approaches for high-throughput clinical validation remains a critical challenge.

In summary, NeoTCRseek is an integrated platform for clinical neoTCR clonotype identification that combines expanded clonotype detection with co-clustering-based TCR prediction. The platform achieves high sensitivity and improved accuracy, supports multi-antigen analysis, and remains compatible with existing adoptive T-cell therapy workflows. This study advances neoTCR clonotype identification for clinical T-cell dynamics monitoring and highlights the need for further integrated approaches to address the complexity of neoTCR clonotype identification.

## Data Availability

Raw sequencing data are not publicly available due to ethical restrictions but may be made available upon reasonable request and subject to approval by the Institutional Review Board of BGI (BGI-IRB). Requests to access the raw sequencing data should be directed to Bin Song, songbin@genomics.cn. Processed TCR clonotype data, along with the NeoTCRseek source code and analysis pipeline, are available in a publicly accessible GitHub repository: https://github.com/songofbin/NeoTCRseek.
